# The impact of negative cognitive bias on NSSI: mediating non-adaptive cognitive emotion regulation strategies

**DOI:** 10.1186/s12912-024-02006-8

**Published:** 2024-05-30

**Authors:** Xuanye Han, Yuhuan Zhang, Dong Chen, Jingyan Sun, Zhixin Di, Zi Yang, Huanchen He

**Affiliations:** 1 Neurosurgery, The Second Affiliated Hospital of Harbin Medical University, 246 Xuefu Road, Harbin, Heilongjiang Province 150000 China; 2 Student Department, The Second Affiliated Hospital of Harbin Medical University, 246 Xuefu Road, Harbin, Heilongjiang Province 150000 China; 3 Helongjiang Nursing College, 246 Xuefu Road, Harbin, Heilongjiang Province 150086 China; 4 Department of Ultrasound Medicine, The Second Affiliated Hospital of Harbin Medical University, 246 Xuefu Road, Harbin, Heilongjiang Province 150000 China; 5 The Second Affiliated Hospital of Harbin Medical University, 246 Xuefu Road, Harbin, Heilongjiang Province 150000 China

**Keywords:** Nursing students, Non-suicidal self-injury, Negative cognitive bias, Non-adaptive cognitive emotion regulation strategies

## Abstract

**Background:**

Individuals may be more likely to engage in NSSI due to negative cognitive bias, while the use of negative emotional regulation mechanisms may further contribute to NSSI. Currently, there is a dearth of studies regarding the correlation among the three variables.

**Method:**

The study employed convenience sampling to collect data via online platforms from a total of 572 college students in Harbin, Heilongjiang Province, China, over the period of January 2024 to February 2024. The questionnaires comprise the Non-Adaptive Cognitive Emotion Srategy Regulation Subscale, the Negative Cognitive Processing Bias Questionnaire, and the NSSI Questionnaire.

**Outcome:**

Negative cognitive bias significantly and directly influences NSSI, as indicated by a beta coefficient of 0.3788 and a confidence interval of [0.2878, 0.4698]. The existence of negative cognitive bias significantly enhances the impact of non-adaptive cognitive emotion control approaches (β = 0.5613, CI [0.4808, 0.6418]). Non-adaptive cognitive emotion regulation strategies showed a significant effect on NSSI, as indicated by a beta coefficient of 0.2033 and a confidence interval of [0.0942, 0.3125]. The non-adaptive cognitive emotion control strategy serves as an intermediary between negative cognitive bias and NSSI, explaining 30.12% of the overall impact.

**In conclusion:**

The results demonstrate that non-adaptive cognitive emotion regulation strategies play a partially moderating role in the relationship between negative cognitive bias and NSSI among nursing students. We emphasize the importance of non-adaptive cognitive emotion regulation strategies, negative cognitive biases, and NSSI among nursing students. In order to reduce the occurrence of NSSI, it is important for schools, families, and teachers to work together closely and implement a well-organized and efficient intervention to protect the mental well-being of nursing students.

## Research background

Non-suicidal self-injury (NSSI) is a significant indicator of both suicide attempts [1] and suicide itself [2], significantly raising the likelihood of suicide by up to sevenfold [3]. It has been acknowledged as a substantial worldwide public health concern [4]. NSSI refers to the intentional act of causing harm or altering body tissue without the intention of taking one's own life. Behaviors such as cutting, scratching, beating, biting, and burning [5, 6], regardless of the perceived severity of the tissue damage, should always be regarded as significant signs of psychological distress [7]. In the past 15 years, there has been an increase in the occurrence of NSSI among youth [8]. According to a recent meta-analysis of 686,672 adolescents and children worldwide, NSSI is the most prevalent behavior, with a lifetime prevalence of 22.1% and a 12-month prevalence of 19.5%. This is followed by suicidal ideation, purposeful self-harm, suicide plots, and attempted suicide [9]. Studies have shown that the occurrence of NSSI in developing nations ranges from 11.5% to 33.8% [10]. According to Swannell [11], approximately 11.5% of young individuals and 20.2% of college students in the overall population have a history of NSSI. The meta-analysis data indicate that the prevalence of NSSI among Chinese college students is 16.20%, making it the third most prevalent mental health condition after sleep disorders and depression [12]. This suggests that NSSI necessitates heightened attention.

The nursing profession encounters several challenges. Nursing students are susceptible to the development of mental and emotional disorders [13] due to the stressors they encounter in clinical and instructional environments, including patient deaths and the psychological strain of working in hospitals. Research indicates that approximately 22% of nursing students are prone to experiencing depression, anxiety, stress, or similar conditions [14]. In China, a study found that 14.9% of nursing students engaged in NSSI [15]. This highlights the importance of paying attention to the self-harming behaviors exhibited by nursing students and emphasizes the need for swift and effective strategies for intervention to be put in place.

Negative cognitive bias is a cognitive characteristic where individuals have a tendency to prioritize the processing of negative information during the information processing phase. The concept encompasses four components: negative attention bias (the inclination to focus on negative information), negative memory bias (the tendency to recall negative information), negative explanation bias (the inclination to interpret ambiguous information in a negative manner), and negative rumination bias (the repetitive contemplation and immersion in negative emotions and feelings) [16, 17]. Currently, there is limited research that elucidates the connection between negative cognitive bias and NSSI. As a result, we incorporate it into a broader scope of negative cognition to better understand the association between negative cognitive bias and NSSI. Studies have demonstrated that adolescents who engage in NSSI have lower levels of cognitive flexibility (i.e., the ability to selectively change cognitive strategies to generate appropriate behavior in constantly changing environments) [18]. According to this information, we put up hypothesis 1: Negative cognitive bias has a positive correlation with NSSI.

Cognitive emotion regulation (CER) pertains to the cognitive processes individuals employ to modify and control their emotions in reaction to particular circumstances [19]. Garnefski [20] categorized nine cognitive emotion regulation strategies, which pertain to an individual's thoughts during and after encountering stressful or threatening situations. These strategies are classified as either "adaptive" or "non-adaptive." Among the many tactics, "non-adaptive strategies" consist of self-blame (holding oneself responsible for the experience), blaming others (holding others responsible for the experience), rumination (obsessive thinking about emotions connected to the experience), and catastrophization (exaggerating the unpleasant parts of the experience).

Negative cognitive bias is not explicitly confirmed by any existing research as being associated with non-adaptive cognitive emotion regulation strategies. Emotion regulation, as postulated by the information processing theory [21] and the process model of emotion regulation [22], is a behavior guided by objectives and characterized by cognitive control mechanisms. Cognitive distortions, such as the erroneous processing of information during regular thought processes, have been linked to the utilization of non-adaptive cognitive emotion regulation strategies, according to prior research [23]. Williams [24] examined the significant effects of adverse memories and negative emotions in a recent study. Yates [25] emphasized the risk factors that are associated with NSSI, including deficient emotion control mechanisms and unfavorable self-schemas. Therefore, we propose hypothesis 2: the propensity to focus on negative cognitive bias is a predictor of employing non-adaptive cognitive emotion regulation strategies.

Prior research has investigated the correlation between NSSI and variables, including "catastrophic" (a non-adaptive regulatory strategy) factors [26, 27]. As per the cognitive-emotion paradigm, individuals who experience strong negative emotions, find it difficult to regulate these feelings, and perceive NSSI as a beneficial method for handling these emotions are more likely to engage in NSSI [28]. According to Nock and Prinstein's theoretical framework, NSSI are maladaptive coping strategies employed to regulate distressing thoughts or emotions through means such as evasion, avoidance, substitution, or direct alteration [29–31]. According to certain research, teenagers who engage in NSSI tend to have more intense negative emotions and struggle with regulating them. This puts them at a higher risk of developing NSSI [32]. Thus, we propose that there is a direct relationship between NSSI and non-adaptive cognitive emotion regulation strategies.

Furthermore, we suggest hypothesis 4: Non-adaptive cognitive emotion regulation strategies serve as a mediator in the association between negative cognitive bias and NSSI. Negative cognitive styles can result in the frequent recollection of negative emotional events. If highly intense negative feelings, like anger, are not alleviated, it can lead to internal violent behaviors such as NSSI and suicidal behavior [33]. During this process, negative cognition, acting as a cognitive factor, can excessively draw individuals' attention to their own bad emotional states, which may be the primary catalyst for engaging in NSSI behavior [34]. Simultaneously, those with negative cognitive styles perceive negative feelings as inappropriate and hold themselves accountable for experiencing such emotions [28]. Consequently, individuals with negative cognitive styles struggle to find efficient methods to handle or prevent intense unpleasant emotions, leading them to resort to avoidance strategies [35]. Prior research has indicated that individuals who employ maladaptive cognitive emotion control techniques are more prone to intensifying their risky behaviors [36].

Overall, there have been few investigations into this issue. Based on a thorough analysis of existing literature, we have formulated four hypotheses on the correlation between negative cognitive bias (NSSI) and non-adaptive cognitive emotion regulation strategies.

1. A tendency toward negative cognitive bias is a strong predictor of NSSI.
2. Negative cognitive bias has a positive effect on non-adaptive cognitive emotion regulation strategies.
3. Non-adaptive cognitive emotion regulation strategies are a strong predictor of NSSI.
4. The relationship between negative cognitive bias and NSSI is influenced by non-adaptive cognitive emotion regulation strategies.

Our research aims to generate innovative intervention strategies for schools and educators to effectively mitigate self-injurious behaviors among nursing students.

## Research objects and methods

### Research objects

Data for the cross-sectional study was collected between January and February 2024, utilizing the online questionnaire platform "Questionnaire Star." The researchers employed convenience sampling to select nursing students from a college located in Harbin, Heilongjiang Province, China. This institution is a public vocational college that operates on a full-time basis. It enrolls more than 7,000 students who are pursuing nursing as their field of study. These students are required to complete a three-year program in order to earn a college degree. The participants in our study were nursing students who willingly took part and were at least 18 years of age. The study did not include nursing students who were on leave of absence, on vacation, or involved in other research.

In order to guarantee the informed permission and voluntary involvement of nursing students, we emphasize the following two aspects at the beginning of the questionnaire: ① This survey is unrelated to everyday performance, and nursing students should participate according to their individual circumstances and available resources. If the submission is completed, it will be regarded as receiving their consent. ②The questionnaire is designed to maintain anonymity and restrict submission to a single instance. Once submitted, the researchers will keep it for safekeeping. If you have any inquiries or if you wish to withdraw, please reach out to the phone number indicated in the questionnaire.

We employed statistical methodologies to determine the appropriate sample size for achieving accuracy. This technique suggests that the sample size should be 5–10 times larger than the number of variables [37]. Studies have demonstrated that factors such as gender, age, grade, location of origin, and family economy have a significant influence on NSSI [38–40]. Therefore, we use these variables as covariates in our research. Hence, we have taken into account a grand total of 14 variables, encompassing gender, age, grade, source of students, average monthly family income, four dimensions of the Non-Adaptive Cognitive Emotion Srategy Regulation Subscale, four dimensions of the Negative Cognitive Processing Bias Questionnaire, and the NSSI Questionnaire. Therefore, a sample size of 140 people was required. In order to account for an expected error rate of 20% in the questionnaires, a sample size of 168 individuals was determined to be sufficient. A grand total of 572 surveys have been distributed. A total of 572 questionnaires were collected, out of which 163 were deemed invalid. After excluding the invalid questionnaires, we analyzed 409 genuine responses. Among these, there were 334 females and 75 men, with an average age of 18.88 ± 0.895 years. The effective response rate was 71.50%. Criteria for exclusion: ① The data has a constant or periodic waveform. The questionnaire was completed in 3 min.

### Research instruments

The questionnaire was segmented into two main portions. The first portion consists of self-reported data, including gender, age, grade level, source of students, and family economic status. The participants completed the digital questionnaire in their native Chinese language, requiring approximately 3–5 min to finish. Part II comprises the following scales:

#### Cognitive Emotion Regulation Questionnaire (CERQ)

Garnefski et al. [41] produced the Chinese version of the cognitive emotion regulation questionnaire, which was later amended by Zhu Xiongzhao et al. [42]. This study employed a non-adaptive cognitive-emotion strategy regulation subscale to examine four dimensions: self-blame, rumination, catastrophizing, and blaming others. The assessment consists of 16 items, each rated on a 5-level scale. The scale ranges from 1 (representing "never") to 5 (representing "always"). The total score can range from 16 to 80 points. As the score on the scale increases, individuals are more inclined to utilize this specific strategy when confronted with bad situations. Example of question: ① I believe I am responsible for the faults; ② I attribute these errors to others; ③ I frequently perceive my experiences as more severe than those of others. The study found that the Cronbach's alpha coefficient for the scale was 0.909. Additionally, each dimension had Cronbach's alpha coefficients of 0.770, 0.930, 0.938, and 0.915, all of which were above the threshold of 0.70. The correlation coefficient between the total score and each item ranged from 0.353 to 0.747 (*P* < 0.01), with a KMO value of 0.900. The Bartlett's sphericity test yielded an approximate value of χ2 = 5029.730 (*P* < 0.001), suggesting strong reliability and validity.

#### Negative cognitive processing bias questionnaire

Zhang Rui's compilation [43] comprises a total of 23 questions, encompassing four distinct dimensions: negative attention bias, negative memory bias, negative explanation bias, and negative rumination bias. This consists of two deception detection questions ("I have never uttered a falsehood" and "I have never experienced any illness, not even a mild cold") and one self-evaluation question regarding one's attitude ("I have responded to this questionnaire with utmost sincerity"). Li Kechi's 4-point self-evaluation scale assigns a value of 1 to indicate complete disagreement, 2 to indicate not quite agreement, 3 to indicate slightly agreement, and 4 to indicate entire agreement. The total score range on this scale is between 20 and 80 points. Example of question: ① I find it effortless to become engrossed in distressing images on television and challenging to redirect my focus; ② I consistently retain a vivid recollection of my errors; ③ I frequently contemplate the reasons behind my perpetual sense of inferiority compared to others. The study found that the Cronbach's alpha coefficient for the overall scale was 0.966. Additionally, the Cronbach's alpha values for each dimension were 0.893, 0.877, 0.891, and 0.903, all of which exceeded the threshold of 0.70. The correlation coefficient between the overall score and each item ranged from 0.641 to 0.843 (*P* < 0.01), with a KMO value of 0.966. The Barrett sphericity test yielded an approximate value of χ^2^ = 6660.118 (*P* < 0.001), suggesting strong reliability and validity.

#### NSSI questionnaire

Wan Yuhui et al. [44] gathered a collection of 12 projects and evaluated them using Li Kechi's 5-level scoring method. The scoring system rates the projects on a scale of 0 to 4, where 0 represents no occurrence, 1 represents infrequent occurrence, 2 represents occasional occurrence, 3 represents frequent occurrence, and 4 represents constant occurrence. The overall score range spans from 0 to 48 points, with higher scores indicating greater severity of NSSI. For example: ① Deliberately etching characters or symbols onto the skin; ② Intentionally biting one's own body; ③ Deliberately tearing off one's own hair. The study found that the Cronbach's alpha coefficient of the scale was 0.984, and the correlation coefficient between the total score and each item ranged from 0.877 to 0.954 (*P* < 0.01). The KMO score was 0.937, and the Bartlett's sphericity test yielded an approximate value of χ^2^ = 9228.135 (*P* < 0.001), showing strong reliability and validity.

### Statistical methodologies

The studies were performed using IBM SPSS statistical software version 24.0. The alpha value for the two-tailed test is set at 0.05, which represents the significance level. Continuous data is typically expressed as the mean value plus or minus the standard deviation. On the other hand, categorical data is usually represented by the number of observations (n) and the corresponding percentage (%). Employ Spearman correlation analysis to assess the correlation between variables. McKinnon's four-step method [45] is employed to examine mediating effects, which must satisfy four distinct criteria: (1) There is a strong correlation between the independent variable (NSSI) and the dependent variable (negative cognitive bias). (2) There is also a strong correlation between the independent variable (NSSI) and non-adaptive cognitive emotion regulation strategies. (3) After controlling for the independent variable (NSSI), there is a strong correlation between the non-adaptive cognitive emotion regulation strategies and the dependent cognitive bias. (4) The indirect correlation coefficient between the independent variable (NSSI) and the dependent variable (negative cognitive bias) through the mediating variable (non-adaptive cognitive emotion regulation strategies) is significant. The initial three phases are evaluated using linear regression equations, with α In set at 0.05 and α Out set at 0.10. The mediating impact was analyzed using the PROCESS macro (Model 4) of SPSS version 3.3. Statistical significance is observed when the 95% confidence interval does not encompass the value of 0.

## Results

### General demographic description

Table 1 displays the sociodemographic attributes of student care. Out of the 409 nursing students that were eligible, 334 of them (81.7%) were females, while 75 of them (18.3%) were males. The mean age of participants is 18.88 years, with a standard deviation of 0.895 years. Out of the total number of nursing students, 314 (76.8%) were in their first year, 91 (22.2%) were in their second year, and 4 (1.0%) were in their third year. 259 individuals, accounting for 63.3% of the total, originate from rural areas, while 150 individuals, representing 36.7%, hail from cities. Within the group of nursing students polled, the majority of families had an average monthly income that fell within the range of less than 3000 yuan and 3001–5000 yuan. Specifically, 36.4% of families have an income below 3000 yuan, while 39.4% have an income between 3001 and 5000 yuan.

**Table 1:** General information statistics of participants (*n* = 409)

Variable		N (%)/M ± SD
Gender		
	male	75(18.3%)
	female	334(81.7%)
Age	18.88 ± 0.895	
Grade		
	Freshman year	314(76.8%)
	Sophomore year	91(22.2%)
	Junior year	4(1.0%)
Source of students		
	rural area	259(63.3%)
	city	150(36.7%)
Average monthly household income		
	< 3000	149(36.4%)
	3001–5000	161(39.4%)
	5001–8000	64(15.6%)
	> 8001	35(8.6%)

### Common method bias

To overcome bias in commonly employed techniques, this study employed Harman univariate analysis to examine the presence of common method bias. Studies have demonstrated that there are 10 factors with eigenvalues exceeding 1. The first common factor explains 33.854% of the variance, which is below 40%. This suggests that the common method bias in this study falls within an acceptable range.

### Correlation analysis between variables

The Table 2 displays the total score of negative cognitive bias as (39.89 ± 13.222) points, the total score of non-adaptive cognitive emotion regulation strategies as (41.49 ± 10.156) points, and the NSSI as (14.81 ± 7.740) points. The presence of negative cognitive bias is strongly and positively associated with non-adaptive cognitive emotion regulation strategies (*r *= 0.547, *p* < 0.01). Additionally, negative cognitive bias is significantly and positively correlated with NSSI (*r* = 0.340, *p* < 0.01). Furthermore, non-adaptive cognitive emotion regulation strategies are significantly and negatively correlated with NSSI (*r* = 0.287, *p* < 0.01).

**Table 2:** Mean, standard deviation, and correlation of all variables (*n* = 409)

Item	Mean ± SD	1	2	3	4	5	6	7	8	9	10	11
1	39.89 ± 13.222	1										
2	9.53 ± 3.453	0.917^**^	1									
3	10.75 ± 3.663	0.937^**^	0.814^**^	1								
4	9.76 ± 3.429	0.918^**^	0.799^**^	0.839^**^	1							
5	9.85 ± 3.641	0.931^**^	0.828^**^	0.836^**^	0.814^**^	1						
6	41.49 ± 10.156	0.547^**^	0.495^**^	0.533^**^	0.485^**^	0.528^**^	1					
7	10.88 ± 3.060	0.351^**^	0.306^**^	0.379^**^	0.283^**^	0.339^**^	0.708^**^	1				
8	12.17 ± 3.386	0.396^**^	0.321^**^	0.424^**^	0.324^**^	0.357^**^	0.655^**^	0.543^**^	1			
9	9.16 ± 3.606	0.500^**^	0.471^**^	0.448^**^	0.469^**^	0.510^**^	0.799^**^	0.386^**^	0.296^**^	1		
10	9.28 ± 3.456	0.418^**^	0.412^**^	0.384^**^	0.389^**^	0.394^**^	0.742^**^	0.300^**^	0.255^**^	0.717^**^	1	
11	14.81 ± 7.740	0.340^**^	0.328^**^	0.299^**^	0.318^**^	0.334^**^	0.287^**^	0.135^**^	0.129^**^	0.336^**^	0.322^**^	1

1. Negative cognitive bias; 2. Negative attention bias; 3. Negative memory bias; 4. Negative explanation bias 5. Negative rumination bias, 6. non-adaptive cognitive emotion regulation strategies; 7. self-blame; 8. reflection; 9. catastrophe; 10. blaming others. 11.NSSI. Dimensions 2, 3, 4, and 5 are part of negative cognitive bias (1); Dimensions 7, 8, 9, and 10 are part of non-adaptive cognitive emotion regulation strategies (6)

(**Indicates *P* < 0.01)

### Examination of the mediating impact of non-adaptive cognitive emotion regulation strategies

Control factors such as gender, age, grade, source of students, and household economy were standardized and employed while examining the mediating influence in this study.

The findings are displayed in Table 3. Negative cognitive bias has a substantial direct effect on NSSI (β = 0.3788, CI [0.2878, 0.4698]). Negative cognitive bias significantly influences non-adaptive cognitive emotion regulation strategies positively (β = 0.5613, CI [0.4808, 0.6418]). Non-adaptive cognitive emotion regulation strategies had a substantial impact on NSSI, with a beta coefficient of 0.2033 and a confidence interval of [0.0942, 0.3125]. Even with mediating factors accounted for, the impact of negative cognitive bias on NSSI remains statistically significant (β = 0.2647, CI [0.1561, 0.3732]).

**Table 3:** Regression analysis of the relationship between various variables

Regression equation		Overall fit index			Regression coefficient significance			
Outcome	Predictor	R	R^2^	F	B	t	LLCI	ULCI
NSSI	Negative cognitive bias	0.3904	0.1524	12.0463 ^a^	0.3788	8.1857^a^	0.2878	0.4698
Non-adaptive cognitive emotion regulation strategies	Negative cognitive bias	0.5801	0.3365	33.9802^a^	0.5613	13.7080^a^	0.4808	0.6418
NSSI	Negative cognitive bias	0.4241	0.1798	12.5602^a^	0.2647	4.7939 ^a^	0.1561	0.3732
	Non-adaptive cognitive emotion regulation strategies				0.2033	3.6623^a^	0.0942	0.3125

^a^Indicates significant at the 0.001 level

**Fig. 1:**
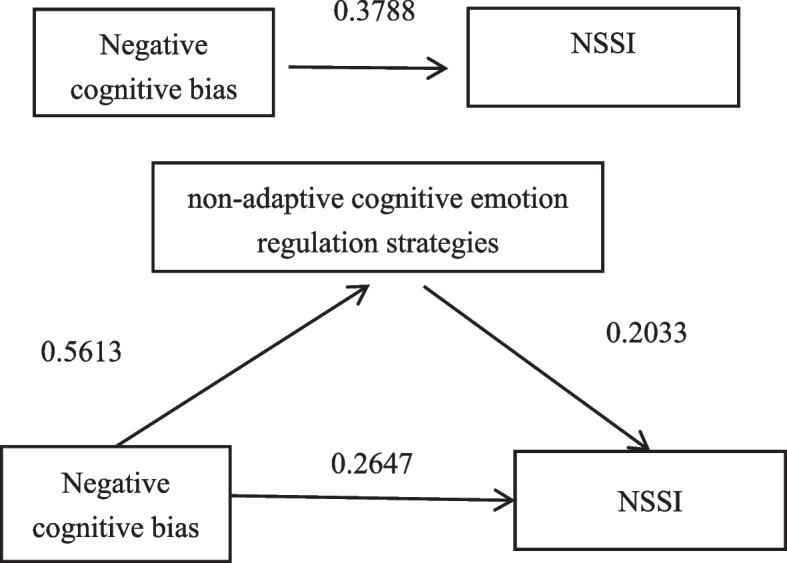
Hypothesized mediation model of the impact of negative cognitive bias on NSSI through non-adaptive cognitive emotion regulation strategies all coefficients are standardized coefficients

Applying the bias-corrected non-parametric percentile Bootstrap method for testing with a bootstrap sample size of 5000 and using a 95% confidence interval without any guidance from zero, we can conclude that non-adaptive cognitive emotion regulation strategies play a mediating role in the relationship between negative cognitive bias and NSSI. The mediating effect explains 30.12% of the overall effect, indicating that non-adaptive cognitive emotion regulation strategies partially mediate the connection between negative cognitive bias and NSSI. Table 4 and Fig. 1 demonstrate the conclusive mediating model. Thus, assumption 4 has been verified.

**Table 4:** Bootstrap analysis of non-adaptive cognitive emotion regulation strategies as mediating variables

Project	Effect size	Boot standard error	LLCI	ULCI	Effect proportion
Mediating effect	0.1141	0.0429	0.0345	0.2029	30.12%
Direct effect	0.2647	0.0552	0.1561	0.3732	69.88%
Total effect	0.3788	0.0463	0.2878	0.4698	100%

## Discussion

### Negative cognitive bias and NSSI

Our study demonstrated that there is a strong correlation between negative cognitive bias and NSSI among nursing students (β = 0.3788, CI [0.2878, 0.4698]). This finding aligns with a recent report by Sorgi, which found that individuals with a history of NSSI exhibit higher levels of negative thinking and negative self-awareness compared to those without a history of NSSI [46]. This is because the presence of a negative processing bias is essential for sustaining negative emotional states, seeking out suicide-related information, triggering suicidal thoughts and behaviors, and raising the probability of suicide [47]. Research has demonstrated that those who possess stronger negative cognitive biases are more inclined to interpret uncertain life circumstances in a pessimistic manner rather than a positive or neutral one. They have a tendency to focus on and retain negative information or thoughts, engage in repetitive thinking, and become deeply absorbed in negative emotions and moods. This phenomenon will prompt individuals to indulge in persistent, prolonged, and recurring pessimistic contemplation over their own selves, emotions, personal issues, and distressing encounters, intensifying negative sentiments and ultimately compelling individuals to resort to NSSI as a means of escaping these intense emotions [48].

### The intermediary function of non-adaptive cognitive emotion regulation strategies

Our work confirms the hypothesis that non-adaptive cognitive emotion regulation strategies have a mediating role in the association between negative cognitive bias and NSSI. This reveals the potential pathways via which negative cognitive bias indirectly influences NSSI. Negative cognitive bias not only directly influences NSSI but also indirectly affects it through non-adaptive cognitive emotion management mechanisms.

The study's findings indicate that negative cognitive bias strongly influences non-adaptive cognitive emotion regulation strategies, as supported by validated hypothesis 2 (β = 0.5613, CI [0.4808, 0.6418]). The detachment from injury hypothesis posits that there exists a detrimental cycle between rumination, a negative cognitive attribute, and bad emotions. Persistent contemplation results in adverse emotional outcomes, and these bad feelings can also generate negative thinking, intensifying the process of contemplation [49]. In contrast, non-adaptive cognitive emotion control methods have a substantial impact on NSSI (β = 0.2033, CI [0.0942, 0.3125]), confirming hypothesis 3. Studies have demonstrated that people who struggle with controlling their emotions are more likely to participate in dangerous activities. As a result, NSSI is sometimes used as a means of regulating or expressing emotions in an effort to diminish or relieve unfavorable emotional encounters [50, 51]. Furthermore, the findings of this study revealed that negative cognitive bias contributed to 69.88% of the overall impact, underscoring the importance of addressing negative cognitive bias in order to accurately detect NSSI in nursing students.

Additionally, we discovered that non-adaptive cognitive emotion regulation strategies had a mediating effect, which accounted for 30.12% of the overall effect. The theory of emotional cognitive evaluation posits that an individual's cognitive assessment of an event or situation can elicit various emotional states and influence subsequent behavior [52]. When nursing students experience negative life events such as academic pressure, interpersonal relationships, punishment, and emotions, these events interact and trigger negative cognitive thinking and behavior [21]. This can result in self-blame and excessive self-criticism, a negative outlook on everything, and being dominated by negative cognitive patterns. Consequently, nursing students may resort to negative coping mechanisms or avoidance when faced with problem-solving [53]. Individuals who frequently employ avoidance methods as a means of dealing with difficult situations generally encounter more intense negative feelings. Currently, nursing students employ NSSI as a means to promptly reduce intense unpleasant or disagreeable feelings within a brief timeframe, resulting in a state of calmness and relaxation [54]. These are potential reasons for the connection between negative cognitive bias, maladaptive cognitive emotion control techniques, and NSSI.

It should be pointed out that we believe that the results of this study may also be applicable to other adolescent groups, for the following reasons:

Adolescence is a crucial period of physical and psychological maturation, during which the brain, behavior, and cognitive systems are not yet fully developed. Individuals are inclined to engage in negative thinking when confronted with negative stressors, which impairs their problem-solving abilities and their capacity to employ effective coping mechanisms. Consequently, they are unable to select appropriate strategies to regulate their emotions, resulting in the buildup of negative emotions and facilitating the onset of NSSI [55, 56]. Nevertheless, it is important to acknowledge that this study specifically examines students. Given the substantial disparities in life experiences and social support between student and non-student populations [55], the generalizability of the research findings to other non-student groups is still a subject of controversy.

## Clinical importance

This study is the first to investigate the connection between non-adaptive cognitive emotion regulation strategies and negative cognitive bias in nursing students and NSSI. Our research has uncovered specific pathways via which negative cognitive bias impacts NSSI, hence enhancing our understanding of the connection between negative cognitive bias and NSSI. Through effectively addressing negative cognitive biases and non-adaptive cognitive emotion regulation strategies, we offer a framework for intervention in nursing NSSI.

Our research indicates that it is crucial to take into account individual negative cognitive biases and emotion management approaches, in addition to focusing on NSSI. When dealing with NSSI, it is crucial to prioritize a comprehensive design approach that emphasizes family support, emotional regulation, and professional assistance [57]. Interventions aimed at preventing self-injury should be developed and implemented within the school setting. School psychologists and workers have a vital role in preventing NSSI [4]. The main focus is on the key processes of NSSI behavior, negative cognitive preference control, and emotion management approaches. Techniques such as mindfulness intervention [58], cognitive behavioral therapy [59], and other methods can be employed to effectively address the negative cognitive bias experienced by nursing students. To encourage emotional expression among nursing students, one can enhance campus cultural activities, establish a platform for sharing viewpoints, arrange group events, offer mental health education sessions, and implement other similar efforts. The objective of these endeavors is to aid nursing students in identifying emotional avoidance, openly expressing their feelings, and improving their skills in emotional regulation. Interpersonal communication refers to the exchange of information and ideas between individuals.

## Conclusion

The outcomes of the study highlight the importance of treating NSSI in nursing students and specifically examine the effects of negative cognitive bias and non-adaptive cognitive emotion regulation mechanisms in this group. Research has indicated that nursing students who have a pessimistic cognitive bias are more likely to employ non-adaptive cognitive emotion regulation strategies, which can result in a higher probability of engaging in NSSI among nursing students.

The research findings underscore the need for nursing supervisors and teachers to promptly identify NSSI, negative cognitive biases, and non-adaptive cognitive emotion regulation strategies. To effectively reduce the occurrence of NSSI in nursing students, it is recommended to employ optimized design and systematic intervention that specifically targets NSSI, negative cognitive bias, and non-adaptive cognitive emotion regulation mechanisms.

## Constraints

Our study has several constraints. Initially, we assessed a single mediating variable. The mediating impact accounts for 30.92% of the total effect. However, when considering the influence of negative cognitive bias on NSSI, there could potentially be additional routes, such as personality traits, coping mechanisms, unpleasant emotions, and various other factors. It is imperative to do additional research in the future to investigate the potential impact of various variables on NSSI in terms of negative cognitive bias. This will enable us to continually enhance our understanding of the relationship between negative cognitive bias and NSSI. Furthermore, it is important to note that this study is cross-sectional, meaning that it is not possible to establish causal relationships between the variables being studied. Moreover, as all the study variables were based on self-reports, there is a possibility of subjective bias. Ultimately, this study employed a convenience design, which limits the extent to which the results may be applied to a broader population. In the future, it will be crucial to utilize more logical sampling methods and select larger and more representative samples to investigate and strengthen the causal connections between the elements discovered in this study.

## Data Availability

The dataset used and/or analyzed in this study can be obtained from appropriate authors upon reasonable request. Author's name and contact information: Zhang Yuhuan, 2802262584@qq.com.
